# Repurposing of antiparasitic drugs: the hydroxy-naphthoquinone buparvaquone inhibits vertical transmission in the pregnant neosporosis mouse model

**DOI:** 10.1186/s13567-016-0317-1

**Published:** 2016-02-17

**Authors:** Joachim Müller, Adriana Aguado-Martínez, Vera Manser, Ho Ning Wong, Richard K. Haynes, Andrew Hemphill

**Affiliations:** Institute of Parasitology, Vetsuisse Faculty, University of Berne, Länggass-Strasse 122, 3012 Bern, Switzerland; Centre of Excellence for Pharmaceutical Sciences, Faculty of Health Sciences G2, North-West University Potchefstroom Campus, 11 Hoffman Street, Potchefstroom, 2531 South Africa

## Abstract

The three anti-malarial drugs artemiside, artemisone, and mefloquine, and the naphthoquinone buparvaquone known to be active against theileriosis in cattle and *Leishmania* infections in rodents, were assessed for activity against *Neospora caninum* infection. All four compounds inhibited the proliferation of *N. caninum* tachyzoites in vitro with IC_50_ in the sub-micromolar range, but artemisone and buparvaquone were most effective (IC_50_ = 3 and 4.9 nM, respectively). However, in a neosporosis mouse model for cerebral infection comprising Balb/c mice experimentally infected with the virulent isolate Nc-Spain7, the three anti-malarial compounds failed to exhibit any activity, since treatment did not reduce the parasite burden in brains and lungs compared to untreated controls. Thus, these compounds were not further evaluated in pregnant mice. On the other hand, buparvaquone, shown earlier to be effective in reducing the parasite load in the lungs in an acute neosporosis disease model, was further assessed in the pregnant mouse model. Buparvaquone efficiently inhibited vertical transmission in Balb/c mice experimentally infected at day 7 of pregnancy, reduced clinical signs in the pups, but had no effect on cerebral infection in the dams. This demonstrates proof-of-concept that drug repurposing may lead to the discovery of an effective compound against neosporosis that can protect offspring from vertical transmission and disease.

## Introduction

*Neospora caninum* is a cyst-forming apicomplexan parasite closely related to *Toxoplasma gondii*. *N. caninum* is one of the most important infectious causes of bovine abortion, stillbirth, and the birth of weak calves, with an economic impact of over 1.3 billion US dollars [1–3]. *N. caninum* infection can also result in birth of clinically healthy, but persistently infected calves transmitting the parasite to the next generation. In addition, *N. caninum* causes neuromuscular disease in dogs, and neosporosis has also been detected in a wide range of other species of livestock and wild animals worldwide.

Possible strategies to limit the economic impact of neosporosis include testing and culling of seropositive animals, discontinued breeding with offspring from seropositive cows, vaccination of susceptible and infected animals, and chemotherapeutical treatment of calves from seropositive cows [4, 5]. However, the most effective option is not always the most economic one [6] and the suitability of any of these options has to be assessed [6, 7].

As is the case for neglected diseases, one option for chemotherapy of neosporosis in cattle is the repurposing of drugs with well-documented antiparasitic activities [8–10]. The most prominent compounds in this context are certainly antimalarial drugs, for which enormous screening campaigns have been initiated, such as the high-throughput screening efforts of St. Jude Children’s Research Hospital (TN, USA), Novartis and GlaxoSmithKline [11–13]. While such large screening campaigns cannot be carried out for *N. caninum,* simply due to the lack of resources, one possibility is to use a piggy-back approach and perform efficacy testing of selected drugs that are already either marketed or in clinical trials for human and/or veterinary use. By screening a larger panel of such compounds, we here report on the results obtained with the three antimalarials artemisone, artemiside, and mefloquine, and the naphthoquinone buparvaquone.

Artemisone and artemiside are artemisinin derivatives [14] that have shown promising efficacy against experimentally induced toxoplasmosis in mice [15]. Moreover, artemisone is highly active against *N. caninum* tachyzoites in vitro [16] and was shown to protect gerbils against cerebral infection [17]. Artemiside exhibits excellent efficacy against malaria in a murine model [18], but has not been tested against neosporosis so far. Mefloquine, one of the most common antimalarials [19], is active in a mouse model of *Schistosoma mansoni* infection [20] and shows promising results in *Echinococcus multilocularis* infected mice [21], but its effect against *N. caninum* has never been assessed. Buparvaquone was also originally developed as an anti-malarial compound [22] and is now commercially available (*Butalex*^*®*^), for use in endemic regions against theileriosis in cattle. In other regions of the world, e.g., the EU, it is, however, not registered. In most cases only a single dose injection is required to cure *Theileria*-infected animals within a few days, with cure rates near to 100% when administered at the early stages of clinical disease [23]. The mode of action of buparvaquone has not been clarified, but there are indications that it blocks the parasite respiratory chain [22], and resistance against buparvaquone has been associated with mutations in the cytochrome b gene of the parasite [24, 25]. Besides exhibiting an outstanding activity against *Theileria* spp. [26, 27], buparvaquone is also active against other protozoan parasites including *Leishmania* spp. [28] and *Babesia* spp. [29]. Moreover, buparvaquone is highly active against *N. caninum* in vitro, and in a non-pregnant mouse model for acute disease we have recently shown that buparvaquone treatment protects mice against infection in the lungs, but not the brain, and prevents clinical signs of acute disease [30].

Here we applied a standardized Balb/c mouse model infected with the *N. caninum* NcSpain-7 isolate [31] to demonstrate that of these four compounds buparvaquone may have the potential to limit vertical transmission in *N. caninum* infected animals without inducing detrimental effects on pregnancy.

## Materials and methods

### Tissue culture media, biochemicals, and drugs

If not stated otherwise, all tissue culture media were purchased from Gibco-BRL (Zürich, Switzerland), and biochemical reagents were from Sigma (St. Louis, MO, USA). Kits for molecular biology were purchased from Qiagen (Hilden, Germany). Buparvaquone was provided by Cross Vetpharm Group Limited (Dublin, Ireland). Mefloquine was kindly supplied by Mepha Pharma AG (Aesch BL, Switzerland). Artemiside was synthesized according to the literature procedure [18] and artemisone was purified and supplied by Cipla Mumbai Ltd.

### Host cell cultivation and parasite cultures

Tachyzoites of the *N. caninum* Nc-Spain7 isolate were cultured and prepared for infection as described [31].

### In vitro efficacy

In vitro efficacies of the compounds were determined using a *N. caninum* beta-galactosidase reporter strain (Nc-beta-gal) and human foreskin fibroblasts (HFF) as host cells. Briefly, HFF were seeded into 96-well-plates, grown to confluence, and infected with 10^3^ Nc-beta-gal-tachyzoites per well in the presence of the compound to be tested or DMSO as a solvent control. After 3 days, medium was removed, and after one wash with PBS, cells were overlaid with 0.1 mL PBS containing Triton-X-100 (0.05%) and chlorophenyl-red-beta-galactoside (Roche, Rotkreuz, Switzerland). Absorption was continuously read at 570 nm using a 96-well-plate spectrophotometer (Versamax, Molecular Devices, Sunnyvale CA) [16]. Host cell toxicity was determined by Alamar blue as described [30].

### Animal experimentation

All protocols involving animals were approved by the Animal Welfare Committee of the Canton of Bern under the license BE115/14. All animals used in this study were handled in strict accordance with practices made to minimize suffering. Female and male BALB/c mice, 8 weeks of age, were purchased from a commercial breeder (Charles River, Sulzberg, Germany), and were maintained in a common room under controlled temperature and a 14 h/10 h light cycle according to the guidelines set up by the animal welfare legislation of the Swiss Veterinary Office (approval No. BE 105/14).

### Assessment of the effects of mefloquine, artemisone, and artemiside on male BALB/c mice infected with *N. caninum* Spain-7

Male Balb/c mice were randomly distributed in four groups of six mice each and subcutaneously infected with 10^5^ tachyzoites of the Nc-Spain7 isolate. At day 2 post infection (pi), one group received corn oil alone (placebo control), the others received mefloquine, artemiside, or artemisone (50 mg/kg/day suspended in 100 µL corn oil) for a period of 6 days by gavage once a day. At day 21 pi, the mice were euthanized. Blood was recovered by cardiac puncture and sera were obtained to assess humoral immune responses. Brains and lungs were removed for subsequent determination of parasite load and stored at −20 °C until further processing.

### Assessment of the effects of buparvaquone on non-pregnant and pregnant BALB/c mice infected with *N. caninum* Spain-7

Fourty-eight female and 24 male BALB/c mice were housed, and pregnancy was achieved after synchronization of oestrus [31]. Subsequently, female mice were randomly distributed in 3 groups of 16 mice each. Mice from group 1 and 2 were subcutaneously infected with 10^5^ tachyzoites of the Nc-Spain7 isolate at mid gestation (days 7 after mating), while mice from group 3 were left uninfected and received a culture medium inoculation [31] At day 2 pi, all mice from group 1 received buparvaquone (50 mg/kg/day suspended in 100 µL corn oil) for a period of 6 days by gavage once a day, while the group 2 mice received corn oil alone. Prior to gavage, the corn oil-drug mixture was heated to 37 °C to enhance solubility of the drug. Group 3 remained untouched. Pregnancy was confirmed between days 15–18 of gestation by weighing, and pregnant mice were then allocated into single cages to give birth on days 20–22 and to rear their pups for an additional 30 days. During this time, those females that had remained non-pregnant were maintained in cages of 3-5 mice. Non-pregnant mice were evaluated for clinical signs of disease twice daily and euthanized in a CO_2_ chamber latest at day 21 post infection. Concerning dams and pups, the litter size, i.e., the number of delivered pups per dam, early pup mortality defined as the number of full-term dead pups from birth until day 2 post partum (pp), post-natal mortality defined as the number of dead pups from day 3 to 30 p.p., and clinical signs were recorded. Surviving dams and pups were euthanized at 30 days p.p. (thus 44 days pi). Blood was recovered by cardiac puncture and sera were obtained to assess humoral immune responses. Brains were removed for subsequent determination of parasite load and stored at −20 °C until further processing. The set-up of the in vivo experiments is shown in Table 1.

**Table 1:** Overview of animal experiments presented in this study

	Exp I	Exp II
Gender	Males	Females
Groups	4, 6 mice per group	3, 16 mice per group
Mating	No	Yes
Challenge	Nc-Spain 7 (10^5^)	Nc-Spain 7 (10^5^), 7 days post mating
Treatment	ARI, ARO, MEF, placebo; days 2–7 pi	BPQ, placebo; days 2–7 pi
Euthanasia	21 days pi	non-pregnant: 21 days pi dams: 30 days after birth, i.e., 44 days pi
Parameters	*N. caninum* load in brains and lungs, serum titer	Number of pups, *N. caninum* brain load, serum titer

Balb/c mice were used throughout the experiment, either males for an in vivo pre-test of artemiside, artemisone or mefloquine (ARI, ARO, MEF; Exp I), or females for assessing the effectiveness of buparvaquone (BPQ) in a pregnant mouse model (Exp. II). pi, post infection with 10^5^ NcSpain7 tachyzoites.

### Analysis of biological samples from in vivo experiments

To quantify the parasite load in brains and lungs, DNA purification was performed employing the DNeasy Blood & Tissue Kit (Qiagen, Basel, Switzerland) according to the standard protocol suitable for animal tissues. The DNA concentrations in all samples were determined using the QuantiFluor dsDNA System (Promega, Madison, Wi., USA) according to the manufacturer’s instructions and adjusted with sterile DNAse free water to 5 ng/µL. Quantification of parasite loads in brains and lungs was performed as described [30, 32]. Serum titers for *N. caninum* were assessed by ELISA as described [33, 34].

### Statistics

Statistical analysis of the parasite burdens in brains was done using the Kruskal–Wallis test followed by Wilcoxon rank-sum-test. Survival analysis of the pups was performed on the corresponding Kaplan–Meier estimator using the log-rank-test. Nominal data were analyzed using the Chi square test. All analyses were performed using the software package R [35].

## Results

### In vitro efficacies of artemisone, artemiside, mefloquine and buparvaquone against *N. caninum* tachyzoites

**Figure 1:**
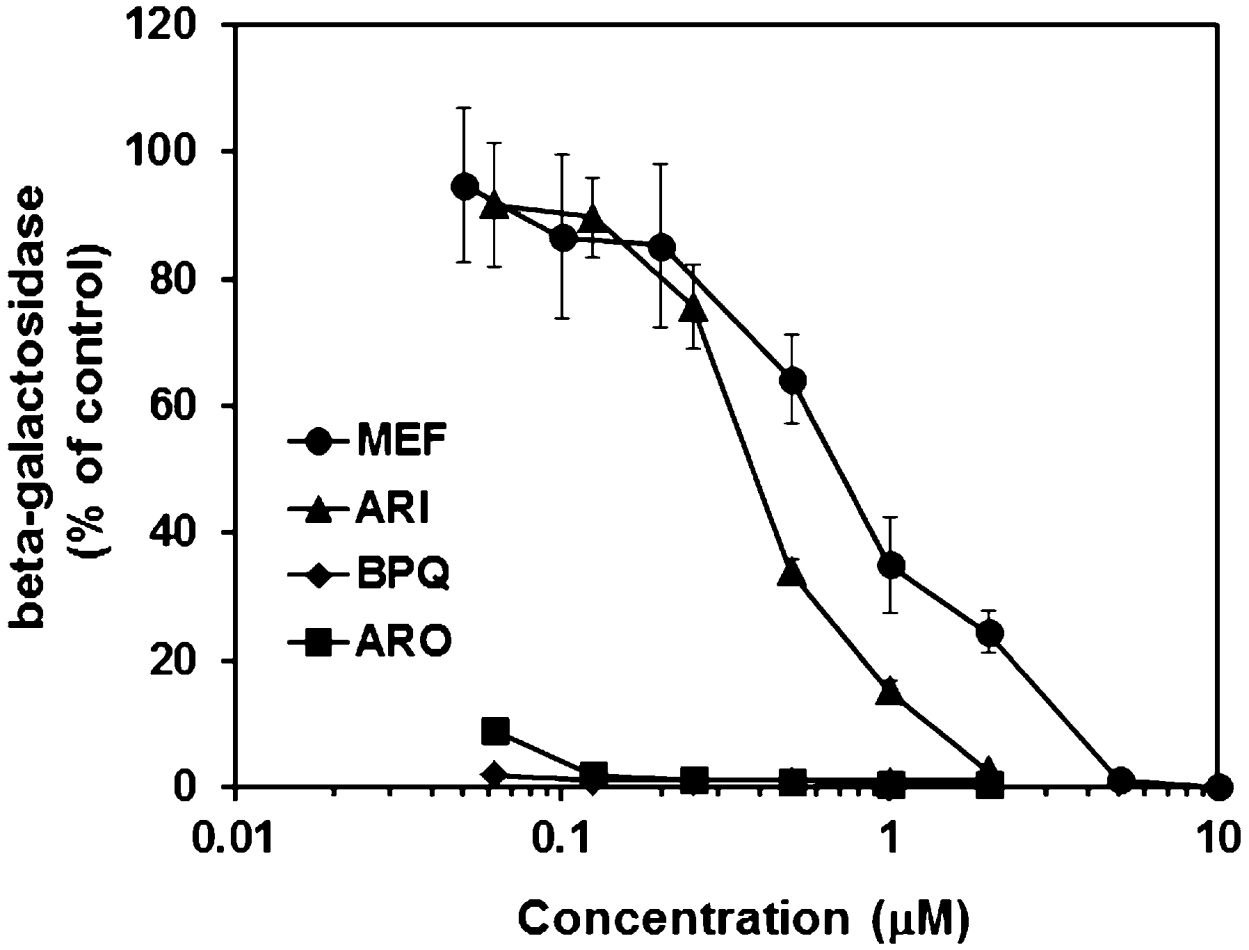
**Inhibition of** ***N. caninum*** **proliferation in vitro.** Human foreskin fibroblasts (HFFs) were grown to confluence in 96-well-plates, treated with the compounds at various concentrations or with DMSO as a solvent control and infected with Nc-beta-gal tachyzoites (10^3^ per well). After 3 days, beta-galactosidase activity was determined. Mean values ± SE are given for four wells relative to the solvent control.

Prior to in vivo studies, the efficacies of artemisone, artemiside, mefloquine, and buparvaquone against the proliferation of *N. caninum* tachyzoites in human foreskin fibroblasts were determined. Mefloquine and artemiside inhibited proliferation with a much higher IC_50_ than artemisone and buparvaquone as visualized by combining the inhibition curves of these four compounds at the same scale (Figure 1). In parallel, cytotoxicity of these four compounds on the human fibroblast host cells was assessed. Of these four compounds, only mefloquine showed a moderate host cell toxicity with an IC_50_ below 5 µM, while the other three compounds exhibited toxicity at concentrations only at concentrations above 5 µM (Table 2).

**Table 2:** In vitro efficacies of the compounds used in this study

Compound	IC_50_ Nc	IC_50_ HFF	Reference
Artemiside	346 (308–388)	>5000	This work
Artemisone	3 (1–13)	>5000	[16]
Buparvaquone	4.9 (4.0–5.9)	>5000	[30]
Mefloquine	941 (775–1141)	3643 (3062–4335)	This work

Human foreskin fibroblasts (HFF) were grown to confluence in 96-well-plates, treated with the compounds at various concentrations or with DMSO as a solvent control, and after 3 days viability was measured using the Alamar blue assay. To determine the inhibition of *N. caninum* (Nc) tachyzoite proliferation (Nc), HFF monolayers were infected with Nc-beta-gal tachyzoites (10^3^ per well). After 3 days, beta-galactosidase activity was determined. IC_50_ values (inhibitory concentration of 50% of the solvent control value) were calculated as described [16] and are given in nM (with 95% confidence intervals).

### In vivo efficacies in a Balb/c mouse model for cerebral *N. caninum* infection

**Figure 2:**
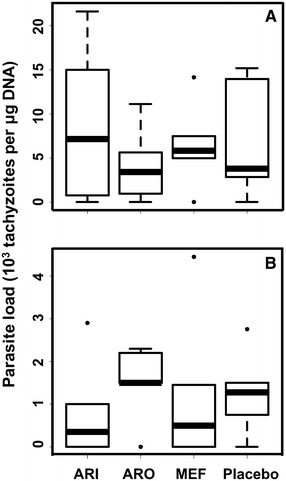
**Parasite load in brains and lungs of mice treated with artemiside, artemisone, or mefloquine.** Male BALB/c mice were infected with Nc-Spain7 tachyzoites and subsequently treated with artemiside (ARI), artemisone (ARO), mefloquine (MEF) in corn oil or corn oil only (Placebo) as detailed in Section “Materials and methods”. The mice were euthanized 3 weeks pi. After euthanasia, brains and lungs were collected and the amount of tachyzoites was determined by quantitative PCR and are presented as box plots (*n* = 6). **A** brains, **B** lungs.

In order to investigate whether artemisone, artemiside and mefloquine also affected *N. caninum* infection, the three anti-malarials were applied in male BALB/c that were infected with 10^5^*N. caninum* Nc-Spain7 tachyzoites. Two days pi, treatment with artemiside, artemisone, or mefloquine (50 mg/g bw in corn oil), or with corn oil alone was initiated and continued over 6 days. All mice were euthanized at day 21 pi. None of the mice showed clinical signs at this stage. The treatments had no effect on parasite loads either in the brain (Figure 2A) or in the lungs (Figure 2B). As a consequence, studies on female mice that had been mated to assess the effects on vertical transmission of *N. caninum* were performed only with buparvaquone.

### Treatment of pregnant mice with buparvaquone protects pups from vertical transmission of *N. caninum*

The results on the effects of buparvaquone treatment in pregnant mice are summarized in Table 3 and Figure 5. Buparvaquone had no significant effects on the reproductive performance as assessed via determination of the pregnancy rate, the litter size and neonatal mortality (Table 3). After the dams had given birth, offspring and dams were maintained for 30 days in order to assess the long-term-impact during the post-natal phase in dams and offspring (Figure 5). Whereas five of eight dams of the placebo control group showed clinical signs and one died before the end of the experiment, none of the buparvaquone treated dams showed clinical signs and none of them died before the end of the experiment (Table 3). In all placebo treated dams and in six of eight buparvaquone treated dams *N. caninum* DNA was detected in the brain by real time PCR (Table 3).

**Table 3:** Effects of buparvaquone treatment on clinical signs, mortality, fertility and cerebral *N. caninum* Nc-Spain7 infection in non-pregnant mice, dams and pups

Parameter	BPQ	Placebo	No treatment
Non-pregnant females			
Number	8	8	7
Clinical signs	0/8	0/8	0/7
Mortality	0/8	0/8	0/7
Seropositive	8/8	8/8	0/7
Nc positive	2/8*	8/8	0/7
Dams and pups			
Number of dams	8	8	8
Clinical signs	0/8^+^	5/8	0/8
Mortality	0/8***	1/8	0/8
Seropositive dams	8/8	8/8	0/8
Nc positive dams	6/8***	8/8	0/8
Pregnancy rate	8/16	8/16	8/15
Total number of pups	46	43	47
Litter size average	46/8***	43/8	47/8
Neonatal mortality^a^	1/46***	2/43	0/47
Postnatal mortality^b^	18/45**	40/41	0/47
Nc positive pups	25/45**	41/41	0/47

BALB/c mice were treated with buparvaquone (BPQ) in corn oil or with corn oil alone (Placebo), infected with Nc-Spain7, or were neither treated nor infected (no treatment), and euthanized as described in Section “Materials and methods”. Adults and surviving pups were tested for the presence of *N. caninum* in their brains by real time PCR. Pups that had died before the end of the experiment were considered as Nc positive. Respective numbers of animals in BPQ and placebo groups were compared by Chi square tests (*** *p* > 0.1, ^+^ *p* < 0.1, * *p* < 0.05, ** *p* < 0.001).

^a^Proportion of pups born dead or that died within the two first days post partum.

^b^Proportion of pups dead from day 3 to 30 pp, considered as Nc positive.

**Figure 3:**
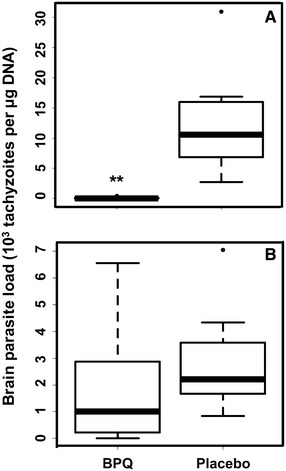
**Cerebral parasite load in non-pregnant and pregnant mice treated with buparvaquone.** BALB/c mice were infected with Nc-Spain7 tachyzoites and subsequently treated with buparvaquone (BPQ) in corn oil or corn oil only (Placebo) as detailed in Section “Materials and methods”. Non-pregnant mice were euthanized 3 weeks pi, dams were euthanized 30 days post partum (pp), thus 44 days pi. After euthanasia, brains were collected and the amount of tachyzoites was determined by quantitative PCR and are presented as box plots (**p < 0.001; Kruskal–Wallis-test). **A** non pregnant mice, **B** dams.

**Figure 4:**
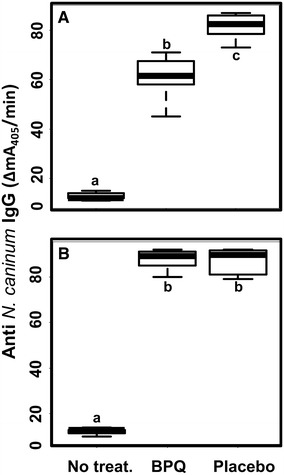
**Anti total** ***N. caninum*** **IgG serum levels.** Balb/c mice were treated as described in Figure 3 (BPQ, Placebo) or left untreated. Serum samples were taken following euthanasia of non pregnant females (**A**) or dams (**B**). ELISA wells were coated with *Neospora caninum* crude antigen. Values are presented as box-plots. Groups labelled with different letters are significantly different (*p* < 0.005; Kruskal–Wallis-test followed by Wilcoxon rank sum test).

None of the non-pregnant mice exhibited clinical signs of neosporosis during 3 weeks following infection (Table 3). In the buparvaquone treated group, the brain parasite load ranged between 0 and 450 tachyzoites per µg DNA as compared to 2700 and 31 000 in the placebo control group. This difference was highly significant (Figure 3A). After 44 days pi, however, in dams that had given offspring, the cerebral parasite loads in buparvaquone-treated dams did not significantly differ from the parasite loads in the placebo groups (Figure 3B). All mice that had been infected were seropositive as assessed by ELISA (Table 3). In the non-pregnant females treated with buparvaquone, the serum titer was, however, not as high as in the placebo group (Figure 4A). In the infected dams, the serum titers were, however, at the same levels (Figure 4B).

**Figure 5:**
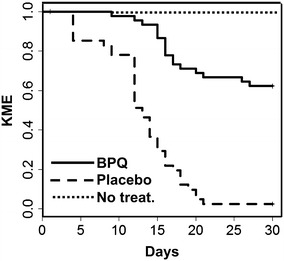
**Offspring from buparvaquone treated dams had a higher survival rate as offspring from placebo treated dams.** Survival curves of pups during 30 days pp of buparvaquone-treated (BPQ, solid line) and corn oil (Placebo, dashed line) treated dams, both of which were infected with Nc-Spain7 isolate of *N. caninum*. As a parameter for survival, the Kaplan–Meier-estimator (KME) is shown. The differences of BPQ and placebo survival curves were highly significant (log-rank-test; *p* < 10^−12^). In the untreated group (No treat.), no pup died (dotted line).

For the offspring, two pups from the placebo group and one from the buparvaquone treated group died immediately after birth. During the next 28 days, 40 of the remaining 41 pups died in the control group, whereas only 18 of 45 in the buparvaquone-treated group died (Table 2). After 30 days, all survivors were tested by PCR for the presence of *N. caninum* in their brains. The last survivor from the placebo group was brain positive, as well as 7 out of 45 surviving pups from buparvaquone treated dams. Thus, mortality and transmission of *N. caninum* to pups were strongly reduced by buparvaquone treatment of the dams. The differences were highly significant (Table 2). This effect was even more apparent after analysis of the survival using the Kaplan–Meier-estimator as survival parameter and by comparing the resulting survival curves using the log-rank-test (Figures 4 and 5).

## Discussion

In order to investigate whether drug repurposing may be a suitable strategy for obtaining suitable chemotherapeutic agents against neosporosis, we have tested four compounds, namely artemiside, artemisone, mefloquine, and buparvaquone, with proven efficacies against apicomplexan parasites in vitro and in mouse models. Three of these four compounds, artemiside, artemisone, and mefloquine did not affect the cerebral parasite load when assessed in a chronic infection model for *N. caninum* infection. This demonstrates that a low IC_50_ value against *N. caninum* tachyzoites obtained in vitro (in the case of artemisone; see also [16]) is not a predictive parameter for good in vivo effects. Moreover, our results are not in line with previous findings concerning effects of artemisone against neosporosis in gerbils [17]. However, in that study artemisone was administered by intraperitoneal injection as a formulation solubilized in DMSO, and the authors used another isolate (NcIS491) from Israel [36], which most likely differs in virulence from the Nc-Spain7 isolate used in this study. In a previous study on experimentally induced murine toxoplasmosis, DMSO solutions of artemisone and artemiside had been applied s.c. [15], but we refrained from this application mode due to potential side effects that is induced by this treatment based on own observations and on previously published results [37]. Oral application of mefloquine, shown to be effective against schistosomiasis [20] and more recently against *Echinococcus* infections in mice [38] did not exhibit any effects against *N. caninum* in this study.

Buparvaquone drastically limits the proliferation of *N. caninum* tachyzoites in vitro. In addition, we have previously shown that administration of buparvaquone protected mice that were infected with a high dosage (2 × 10^6^) of *N. caninum* tachyzoites of the Nc-Liverpool isolate against acute neosporosis [30]. In the present study, by using a 20 times reduced infection dose (10^5^ tachyzoites), which does not lead to acute symptoms but to chronic infection followed by vertical transmission in dams [31], we demonstrate that in the short term, buparvaquone reduces the cerebral parasite load– as seen in the non-pregnant mice that were euthanized at 3 weeks pi. However, in the longer term, as seen in the dams that were euthanized 30 days after birth, that is, 44 days pi, no reduction in the cerebral parasite load was evident. This confirms earlier observations that buparvaquone is parasiticidal against *N. caninum* only after long term treatment [30]. Thus, 6 days of treatment is too short to eradicate the parasite from the dams, but the action of the drug during this short time span is sufficient to consistently impair vertical transmission to the offspring as evidenced by a significantly lower cerebral parasite load and a lower mortality in the offspring. Under the conditions in which we run our in vivo experiments (SPF animals, controlled access, qualified personnel), in uninfected, untreated animals, postnatal mortality does not occur. In the vertical transmission model [31], postnatal mortality occurs only upon dose dependent infection of the dams. Pups of infected dams die within 3 weeks; pups of not infected dams survive. The model is reproducible as shown in a more recent study [39].

In conclusion, this study shows that repurposing of established drugs facilitates the identification of compounds that may be applied for treatment of neosporosis, thus avoiding a long and costly drug development process. Other examples of potentially interesting compounds identified earlier through this avenue, and providing interesting results both in vitro and in vivo are miltefosine [40] and compounds belonging to the class of bumped kinase inhibitors (BKIs) that act against calcium-dependent protein kinase 1, a target that is found in most apicomplexans [32, 39]. BKIs were originally developed to combat malaria [41]. Buparvaquone is already in use against *Theileria* infections in cattle in endemic countries. Thus, it would be of interest to determine the incidence of neosporosis in buparvaquone treated cattle in those regions as compared to a control population that has not been treated. Moreover, studies in cattle will show whether buparvaquone treatments are able to eradicate *N. caninum* from the mother cows or whether they reduce the parasite to a low level chronic infection which may reactivate as soon as drug treatment is withdrawn. After establishment of a suitable treatment scheme, the effects on vertical transmission in cattle could be determined.
